# Real-Time Prescription Benefit Tool Adoption Among US Hospitals

**DOI:** 10.1001/jamahealthforum.2024.3181

**Published:** 2024-10-18

**Authors:** Matthew J. Klebanoff, Pengxiang Li, Paula Chatterjee, Jalpa A. Doshi

**Affiliations:** 1 Division of General Internal Medicine, Perelman School of Medicine at the University of Pennsylvania, Philadelphia; 2 Leonard Davis Institute of Health Economics, University of Pennsylvania, Philadelphia

## Abstract

This cross-sectional study examines real-time prescription benefit tool adoption among US hospitals overall and by hospital and community characteristics.

## Introduction

Real-time prescription benefit tools (RTBTs) display out-of-pocket drug cost estimates in the electronic health record (EHR) at the point of prescribing.^1^ RTBTs can help lower out-of-pocket drug spending.^2^ Since 2021, the Centers for Medicare & Medicaid Services have required Part D plan sponsors to implement RTBTs that can be incorporated into EHRs.^3^ However, health care facilities are not mandated to adopt RTBTs. Hospitals that serve patients who are economically and socially marginalized may be less likely to adopt new health information technology.^4^ This cross-sectional study estimated RTBT adoption among US hospitals overall and across different types of hospitals and hospitals serving different patient populations.

## Methods

The University of Pennsylvania institutional review board exempted this study from review because it was non–human participant research. This study followed the STROBE reporting guideline. We estimated prevalence of RTBT adoption using the American Hospital Association 2022 Information Technology Supplement (eMethods 1 in Supplement 1). Hospitals were deemed RTBT adopters if EHRs included real-time prescription benefit information for all or almost all payers or a limited set of payers (eMethods 2 in Supplement 1). RTBT adoption was compared across hospital characteristics and 2 measures of safety-net hospital status: Medicaid share of discharges and uncompensated care as a share of operating expenses (eTable in Supplement 1). Adoption was compared across county-level health and sociodemographic characteristics from the American Community Survey and County Health Rankings. Inverse probability weighting was used to account for nonresponse (eMethods 3 in Supplement 1). Analyses were conducted from October 2023 to August 2024. χ^2^ Tests were performed using Stata, version 18.0. Two-sided *P* < .05 was considered significant.

## Results

**Figure 1.**
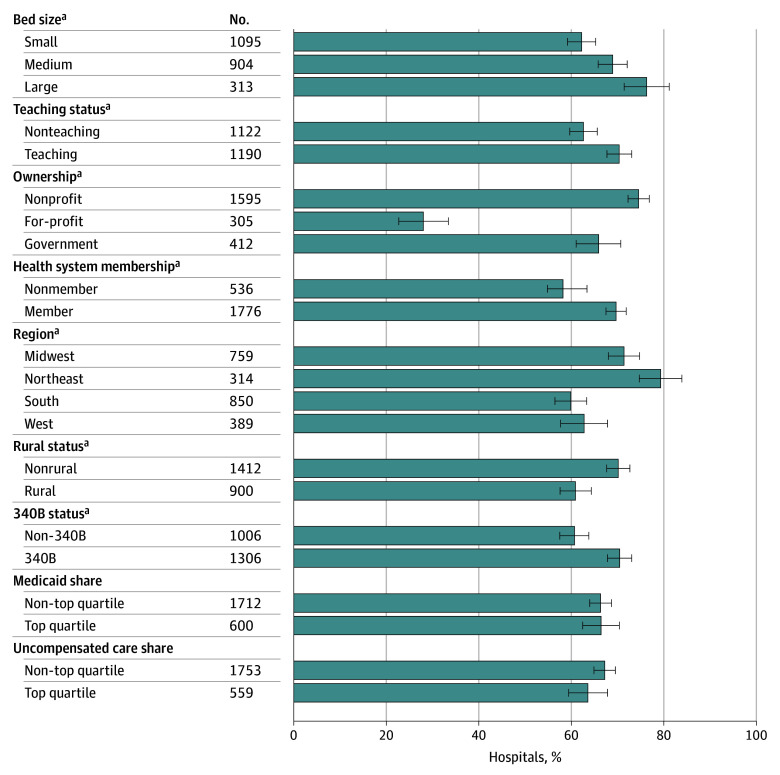
Real-Time Prescription Benefit Tool Adoption by Hospital Characteristics Percentages are weighted. Error bars indicate 95% CIs. ^a^*P* < .001.

**Figure 2.**
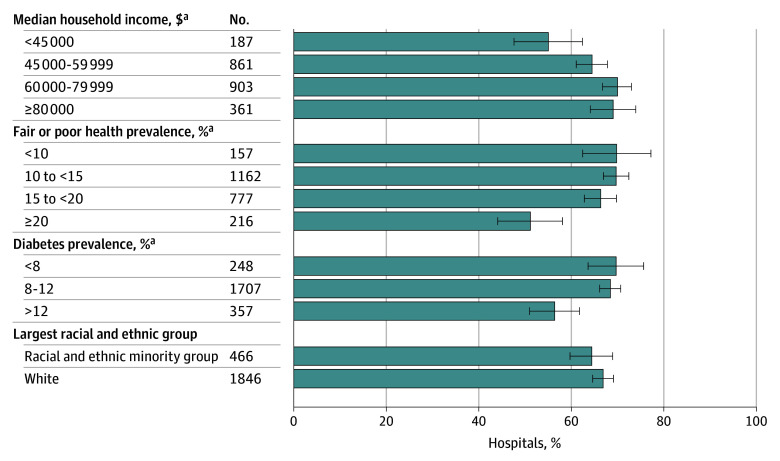
Real-Time Prescription Benefit Tool Adoption by County-Level Community Characteristics Race and ethnicity were ascertained by self-report in the American Community Survey and included in the analysis because racial and ethnic minority populations disproportionately face a high burden of chronic diseases. Counties were classified as including mostly White, non-Hispanic individuals or mostly racial or ethnic minority individuals, as this analysis involved drawing comparisons between discrete categories at the county level. The racial and ethnic minority group included survey respondents who self-identified as African American or Black, American Indian or Alaska Native, Asian, Hispanic or Latino, Native Hawaiian or Other Pacific Islander, or multiracial. Percentages are weighted. Error bars indicate 95% CIs. ^a^*P* < .001.

The analysis included 4145 acute care hospitals, of which 2312 (55.8%) responded to the RTBT item. In weighted analyses, 67.9% reported having an RTBT and 76.1% of RTBTs provided information for all or almost all payers. RTBT adoption was least prevalent (shown with 95% CIs) among hospitals that were small vs large (62.2% [59.2%-65.2%] vs 76.3% [71.4%-81.1%]); nonteaching vs teaching (62.6% [59.6%-65.6%] vs 70.4% [67.7%-73.1%]); for profit vs nonprofit (28.0% [22.7%-33.4%] vs 74.5% [72.3%-76.8%]); nonmembers of health systems vs members (59.1% [54.8%-63.4%] vs 69.7% [67.5%-71.9%]); Southern vs Northeastern (59.9% [56.4%-63.3%] vs 79.3% [74.6%-83.9%]); rural vs nonrural (60.9% [57.6%-64.3%] vs 70.1% [67.6%-72.6%]); or 340B nonparticipants vs participants (60.7% [57.5%-63.8%) vs 70.4% [67.8%-73.1%]) (*P* < .001 for all) (Figure 1). RTBT adoption was also least prevalent (shown with 95% CIs) among hospitals in counties with the lowest median household income vs highest income (55.0% [47.6%-62.4%] vs 69.1% [64.2%-74.0%]); highest prevalence of fair or poor health vs lowest prevalence (51.1% [44.1%-58.1%] vs 69.8% [62.4%-77.2%]); or highest prevalence of diabetes vs lowest prevalence (56.4% [51.0%-61.8%] vs 69.7% [63.7%-75.7%]) (*P* < .001 for all) (Figure 2).

## Discussion

In this sample, 67.9% of hospitals adopted RTBTs. However, RTBT adoption was less common in hospitals that were small, for profit, nonaffiliated with health systems, rural, and in counties with lower household income and worse health measures. While considerable attention has focused on optimizing implementation of RTBTs within clinical workflows,^1,5^ this study highlights a need to ensure these tools reach populations that may benefit most. Some findings, such as the markedly low use of RTBTs at for-profit hospitals, may inform future strategies to encourage RTBT adoption. Recognizing the potential of this technology, Congress has included RTBTs in the definition of a qualified EHR, which could enable development of regulatory incentives to encourage RTBT implementation.^6^

Limitations include nonresponse, a single-item assessment of RTBT adoption, and a focus on hospitals. The survey did not define RTBTs, allowing for variable interpretation. Nonetheless, this study provided, to our knowledge, the first national estimate of RTBT adoption and found potential disparities in access. Future research should identify barriers to installing RTBTs among nonadopting hospitals and examine downstream use of these tools by prescribing clinicians.

## Notes

Supplement 1. **eMethods 1.** Description of Data Sources **eMethods 2.** Description of AHA IT Supplement and RTBT Item
eReference
**eMethods 3.** Creation of Inverse Probability Weights to Account for Non-Response **eTable.** Description of Variables

Supplement 2.
Data Sharing Statement
