# Supplementary material for: Real-Time Prescription Benefit Tool Adoption Among US Hospitals
Source: JAMA Health Forum. 2024 Oct 18;5(10):e243181. doi: 10.1001/jamahealthforum.2024.3181 (PMC11581665; doi:10.1001/jamahealthforum.2024.3181)
Supplement: Supplement 1. — eMethods 1. Description of Data Sources eMethods 2. Description of AHA IT Supplement and RTBT Item eReference eMethods 3. Creation of Inverse Probability Weights to Account for Non-Response eTable. Description of Variables [file jamahealthforum-e243181-s001.pdf]

Supplementary Online Content

Klebanoff MJ, Li P, Chatterjee P, Doshi JA. Real-time prescription benefit tool adoption among US hospitals. *JAMA Health Forum*. 2024;5(10):e243181.
doi:10.1001/jamahealthforum.2024.3181

eMethods 1. Description of Data Sources
eMethods 2. Description of AHA IT Supplement and RTBT Item
eReference
eMethods 3. Creation of Inverse Probability Weights to Account for Non-Response
eTable. Description of Variables

This supplementary material has been provided by the authors to give readers additional information about their work.

© 2024 Klebanoff MJ et al. *JAMA Health Forum*.

eMethods 1. Description of data sources

1. American Hospital Association Annual Survey: The American Hospital Association (AHA) Annual Survey gathers information from thousands of hospitals in the U.S. The survey collects information regarding hospital characteristics, services, financials, and workforce. Participation in the survey is voluntary, but a majority of U.S. hospitals respond to the survey each year. Data from the 2022 AHA Annual Survey were included in this analysis. Additional information on this source can be found in the eTable or at this link: https://www.ahadata.com/aha-data-resources.

2. American Hospital Association Information Technology Supplement Survey: The AHA Information Technology (IT) Supplement Survey is a supplement to the AHA Annual Survey. The survey collects information regarding each hospital's IT capabilities and workforce. Each hospital is invited to send the IT Supplement to the person most knowledgeable about the hospital's IT capabilities—typically a chief information officer. Data from the 2022 AHA IT Supplement were included in this analysis. Additional information on this source can be found in the eTable or at this link: https://www.ahadata.com/aha-data-resources.

3. Medicare Cost Reports: The Medicare Cost Reports are compiled by the RAND Corporation and comprise a dataset of hospital characteristics and finances. Hospitals that receive Medicare payments submit an annual cost report, and RAND has transformed these data into calendar year formatting, which were used in this study. Additional information on this data source can be found in the eTable or at this link: https://www.hospitaldatasets.org/

4. 340B Office of Pharmacy Affairs Information System: The Health Resources and Services Administration (HRSA) provides a searchable online list of 340B-covered entities through the 340B Office of Pharmacy Affairs Information System (340B OPAIS). Data for 2022 were extracted from the 340B OPAIS. Additional information on this source can be found in the eTable or at this link: https://340bopais.hrsa.gov/

5. County Health Rankings: The County Health Rankings (CHR) are a data resource compiled by the University of Wisconsin Population Health Institute and the Robert Wood Johnson Foundation. The CHR gathers county-level information on health outcomes and local factors that affect population health (e.g., health behaviors and environmental factors). The CHR gathers this information from a variety of sources, including the National Center for Health Statistics, the Behavioral Risk Factor Surveillance System, the Area Health Resource File, and the American Community Survey. Data were extracted from the 2023 CHR, which included county-level measures from 2020. Additional information on this source can be found in the eTable or at this link: https://www.countyhealthrankings.org/.

6. American Community Survey: The American Community Survey (ACS) is an annual survey conducted by the U.S. Census Bureau. The ACS gathers information regarding social, economic, and demographic characteristics of communities in the U.S. Five-year estimates for race/ethnicity and median household income representing 2017-2021 were extracted from the ACS. Additional information on this source can be found in the eTable or at this link: https://www.census.gov/programs-surveys/acs.

© 2024 Klebanoff MJ et al. *JAMA Health Forum*.

eMethods 2. Description of AHA IT Supplement and RTBT item

The AHA IT Supplement is sent to each acute care general medical and surgical hospital. The chief executive officer at each hospital receives the survey and distributes it to whomever in the organization is most knowledgeable (e.g., a chief information officer).[1]

The 2022 AHA IT Supplement Survey contained an item regarding real-time prescription benefit tools. This item is included below:

19. *Does your EHR integrate health insurer real-time prescription benefit information?*
    a. ☐ Yes, for all or almost all payers
    b. ☐ Yes, for a limited set of payers
    c. ☐ No
    d. ☐ Don't know

The IT Supplement did not include any definition of "real-time prescription benefit information." Hospitals that reported that their EHR integrated health insurer real-time prescription benefit information "for all or almost all payers" or "for a limited set of payers" were considered RTBT-adopters. When we calculated the overall percentage of RTBT adopters, the numerator included all RTBT-adopters, and the denominator included all respondents to this item (including those who responded "No" or "Don't know"). We suspect that hospitals that reported "Don't know" were non-adopters, as the decision to implement an RTBT requires a conscious decision and effort by health IT professionals; individuals familiar with their hospital's IT capabilities should thus know whether their hospital has implemented an RTBT.

eReference:

1. Jha AK, DesRoches CM, Campbell EG, et al. Use of Electronic Health Records in U.S. Hospitals. *N Engl J Med*. 2009;360(16):1628-1638. doi:10.1056/NEJMsa0900592

© 2024 Klebanoff MJ et al. *JAMA Health Forum*.

eMethods 3. Creation of inverse probability weights to account for non-response

Our sample included 4,145 acute care hospitals that responded to the 2022 AHA Annual Survey. Only a subset of respondents to the Annual Survey responded to the RTBT adoption question in the IT Supplement Survey. To account for this non-response, we constructed a logistic regression model where the outcome was response to the IT Supplement RTBT item. Predictors included hospital characteristics (bed size, teaching status, ownership, health system membership, region, rurality, presence of primary care department or outpatient practices, and 340B status) and county characteristics (median household income, fair or poor health prevalence, diabetes prevalence, and race/ethnicity). Using this model, a predicted probability of being a responder vs. non-responder was generated for each hospital, and inverse probability weights were applied to all respondents to the IT Supplement RTBT survey item.

© 2024 Klebanoff MJ et al. *JAMA Health Forum*.

eTable. Description of variables

Data Source	Variable	Year	Variable for Linkage	Notes
AHA Annual Survey	Beds	2022	n/a	
	Teaching status (teaching, non-teaching)	2022	n/a	Hospitals were classified as teaching hospitals if they 1) reported having one or more Accreditation Council for Graduate Medical Education accredited programs, or 2) reported a medical school affiliation to the American Medical Association, or 3) reported membership in the Council of Teaching Hospital of the Association of American Medical Colleges (COTH), or 4) the ratio of full-time medical and dental residents and interns to the number of hospital beds was greater than or equal to 0.25.
	Ownership (non-profit, for-profit, government)	2022	n/a	
	Rural	2022	n/a	Hospitals that reported a core-based statistical area code of “rural” or “micropolitan” were classified as “rural.”
	Health system member	2022	n/a	
	Primary care or outpatient departments	2022	n/a	Hospitals were classified as having primary care or outpatient departments if they 1) reported having a primary care department affiliated with the hospital, health system, or joint venture, or 2) reported having hospital-based outpatient care center/services affiliated with the hospital, health system, or joint venture, or 3) reported having a freestanding outpatient care center affiliated with the hospital, health system, or joint venture.
AHA IT Supplement	Real-time prescription benefit tool	2022	n/a	See eMethods 2 for additional information regarding this variable.
Medicare Cost Reports	Medicaid share	2022	Medicare provider number	Medicaid share indicates the share of Medicaid inpatient discharges out of total discharges. Hospitals were classified as being in the top quartile of Medicaid share within their state versus all other hospitals.

© 2024 Klebanoff MJ et al. *JAMA Health Forum*.

Data Source	Variable	Year	Variable for Linkage	Notes
	Uncompensated care	2022	Medicare provider number	Uncompensated care indicates the sum of charity care and bad debt as a fraction of operating expenses. Hospitals were classified as being in the top quartile of uncompensated care within their state versus all other hospitals.
340B Office of Pharmacy Affairs Information System	340B status	2022	Medicare Provider ID	A hospital was deemed a 340B hospital if it had active 340B status at any point in 2022.
County Health Rankings	Fair or poor health prevalence	2020	FIPS County Code	We used 2023 County Health Rankings data. This variable includes data from the Behavioral Risk Factor Surveillance System in 2020.
	Diabetes prevalence	2020	FIPS County Code	We used 2023 County Health Rankings data. This variable includes data from the Behavioral Risk Factor Surveillance System in 2020.
American Community Survey	Median household income (2021 $)	2017-2021	FIPS County Code	
	Race/ethnicity	2017-2021	FIPS County Code	Race and ethnicity were ascertained by self-report in the American Community Survey and included in this analysis because racial and ethnic minority populations disproportionately face a high burden of chronic diseases. Counties were classified as including mostly White, non-Hispanic individuals or mostly racial or ethnic minority individuals, as this analysis involved drawing comparisons between discrete categories at the county level. The racial and ethnic minority group included survey respondents who self-identified as African American or Black, American Indian or Alaska Native, Asian, Hispanic or Latino, Native Hawaiian or Other Pacific Islander, or multiracial.

© 2024 Klebanoff MJ et al. *JAMA Health Forum*.
