# Supplementary material for: Real-Time Prescription Benefit Tool Adoption Among US Hospitals
Source: JAMA Health Forum. 2024 Oct 18;5(10):e243181. doi: 10.1001/jamahealthforum.2024.3181 (PMC11581665; doi:10.1001/jamahealthforum.2024.3181)
Supplement: Supplement 2. — Data Sharing Statement [file jamahealthforum-e243181-s002.pdf]

Data Sharing Statement

Klebanoff. Real-Time Prescription Benefit Tool Adoption Among US Hospitals. *JAMA Health Forum*. Published October 18, 2024. doi:10.1001/jamahealthforum.2024.3181

Data
Data available: No

Additional Information
Explanation for why data not available: We are unable to publicly share American Hospital Association survey data.
